# Interactive mapping of language and memory with the GE2REC protocol

**DOI:** 10.1007/s11682-020-00355-x

**Published:** 2020-08-06

**Authors:** Sonja Banjac, Elise Roger, Emilie Cousin, Marcela Perrone-Bertolotti, Célise Haldin, Cédric Pichat, Laurent Lamalle, Lorella Minotti, Philippe Kahane, Monica Baciu

**Affiliations:** 1 Univ. Grenoble Alpes, CNRS LPNC UMR 5105, F-38000 Grenoble, France; 2 Univ. Grenoble Alpes, UMS IRMaGe CHU Grenoble, F-38000 Grenoble, France; 3 Univ. Grenoble Alpes, GIN, Synchronisation et modulation des Réseaux Neuronaux dans l’Epilepsie’ and Neurology Department, F-38000 Grenoble, France

**Keywords:** Language, Memory, fMRI mapping, Cognitive interaction

## Abstract

Previous studies have highlighted the importance of considering cognitive functions from a dynamic and interactive perspective and multiple evidence was brought for a language and memory interaction. In this study performed with healthy participants, we present a new protocol entitled GE2REC that interactively accesses the neural representation of language-and-memory network. This protocol consists of three runs related to each other, providing a link between tasks, in order to assure an interactive measure of linguistic and episodic memory processes. GE2REC consists of a sentence generation (GE) in the auditory modality and two recollecting (2REC) memory tasks, one recognition performed in the visual modality, and another one recall performed in the auditory modality. Its efficiency was evaluated in 20 healthy volunteers using a 3T MR imager. Our results corroborate the ability of GE2REC to robustly activate fronto-temporo-parietal language network as well as temporal mesial, prefrontal and parietal cortices in encoding during sentence generation and recognition. GE2REC is useful because it: (a) requires simultaneous and interactive language-and-memory processes and jointly maps their neural basis; (b) explores encoding and retrieval, managing to elicit activation of mesial temporal structures; (c) is easy to perform, hence being suitable for more restrictive settings, and (d) has an ecological dimension of tasks and stimuli. GE2REC may be useful for studying neuroplasticity of cognitive functions, especially in patients with temporal lobe epilepsy who show reorganization of both language and memory networks. Overall, GE2REC can provide valuable information in terms of the practical foundation of exploration language and memory interconnection.

**Electronic supplementary material:**

The online version of this article (10.1007/s11682-020-00355-x) contains supplementary material, which is available to authorized users.

## Introduction

It is suggested that the base of proper cognitive functioning is the dynamic interaction between different neuropsychological domains (Kellermann et al. 2016). Specifically, there is growing evidence suggesting that memory and language influence each other more than previously thought (Huettig and Janse 2016; Moscovitch et al. 2016; Vogelzang et al. 2017). In the present study we propose a protocol that would allow mapping of the neural representations of the joint language-and-memory network, focusing on autobiographical memory, where the link with language is still under investigation. Several categories of arguments underlie this interaction. For instance, it has been shown that language influences the formation of memories and remembering (Larsen et al. 2002; Marian and Neisser 2000), while memory functioning can manifest through language production (Park et al. 2011). Second, it was suggested that there are brain systems commonalities between these functions (Ullman 2004) and that lexical-semantic aspects of language are highly dependent on the declarative memory system, which is accomplished through the hippocampus (Duff and Brown-Schmidt 2012). The central regions of episodic memory represented by lateral and medial temporal structures, notably the hippocampus and entorhinal cortex, indeed have important connections with the language structures such as temporal association cortex, temporal pole, prefrontal cortex and parietal association cortex (Duvernoy et al. 2013; Tracy and Boswell 2008). Moreover, this interaction can be maintained by certain fibers that support both functions such as the uncinate fascicle (Diehl et al. 2008; Duffau et al. 2014; McDonald et al. 2008) and fronto-occipital fascicle (Duffau et al. 2014; McDonald et al. 2008; Moritz-Gasser et al. 2013).

Furthermore, there are disorders such as post-stroke aphasia (Schuchard and Thompson 2014) or conditions that cause auditory hallucinations (Ćurčić-Blake et al. 2017) in which language regions are impaired, but the symptoms also manifest in the memory domain. Nevertheless, this dynamical language-and-memory relation is most apparent in temporal lobe epilepsy (TLE) that represents 70–80% of epilepsy in adults (Jaimes-Bautista et al. 2015) and is characterized by seizures induced by a regional dysfunction, the epileptic zone (EZ), located in temporal regions. As language and memory networks integrative hubs mainly stem from the left temporal lobe (Battaglia et al. 2011), TLE patients show both naming (Bartha-Doering and Trinka 2014), verbal and long-term memory (Bell et al. 2011; Tramoni-Negre et al. 2017) deficits, more so if the EZ is located mesially (Alessio et al. 2006; Davies et al. 1998; Perrone-Bertolotti et al. 2012; Zalonis et al. 2017). Generally, studies with TLE patients show that the hippocampus has a vital role in retrieving lexically and semantically associated words (Bonelli et al. 2011; Hamamé et al. 2014) and that it is active during language comprehension tasks, as well as its neighboring structures (Meyer et al. 2005). Importantly, hippocampal theta oscillations were associated with lexical-semantic processing (Piai et al. 2016; Pu et al. 2020), leading to the proposal to incorporate this structure into language network (Covington and Duff 2016).

TLE patients are often refractory to drugs and surgical removal of the EZ is the only curative option (Schoenberg et al. 2011; Téllez-Zenteno et al. 2005). Since surgery affects the temporal regions which are crucial for language and memory, the intervention is preceded by detailed preoperative mapping (Baxendale et al. 2006; Drane and Pedersen 2019; Hamberger 2015; Helmstaedter et al. 2003; Sherman et al. 2011) which can be effective in predicting the postoperative decline (Bonelli et al. 2010; Massot-Tarrús et al. 2019; Rosazza et al. 2013). Importantly, it was shown that language fMRI activation can predict verbal memory postoperative outcomes (Binder et al. 2008, 2010; Labudda et al. 2010). This leads to the conclusion that these functions should be assessed in interplay. In addition to more thorough surgical mapping, the benefit of this interactive assessment would be to better understand and predict brain plasticity. Namely, inter and intra-hemispheric cerebral reorganization (Baciu and Perrone-Bertolotti 2015; Berl et al. 2014; Cousin et al. 2008; Dupont 2000; Powell et al. 2007; Rosenberger et al. 2009; Sidhu et al. 2013) in TLE patients can arise due to chronic epilepsy and surgery. The important point is that the reorganization of language can depend on regions that have not classically been considered a part of that network (Tracy and Boswell 2008) such as the hippocampus (Baciu and Perrone-Bertolotti 2015). This could not be captured in the presurgical assessment that relies only on the assessment of this function. Finally, this interactive framework can lead to a unified neurocognitive model filling the gap in the present models (Duffau et al. 2014; Hickok and Poeppel 2007; Indefrey and Levelt 2004; Price 2012; Ullman 2004) that, although comprehensive, rarely consider cognitive domains interaction.

Even though fMRI is currently regarded as an efficient tool for preoperative assessments of cortical regions for the purpose of resection optimization (Abbott et al. 2010; Binder 2011; Sabsevitz et al. 2003), there is no consensus for the most appropriate protocol and paradigm to determine language and memory brain lateralization and localization (Benjamin et al. 2018; Perrone-Bertolotti et al. 2015). Certain authors have proposed fMRI protocols that encompassed both functions in adults (Aldenkamp et al. 2003; Deblaere et al. 2002). However, they examined these two functions separately, concluding their interconnection afterwards. Also, the protocols’ ecological validity is often neglected (Mayer and Murray 2003), with tasks being far from functioning in real-life situations that presurgical assessment is meant to conserve.

Brain activation observed during language and memory mapping is largely determined by the nature of the task (Baciu and Perrone-Bertolotti 2015; Bradshaw et al. 2017). Generally, language tasks should map a network encompassing inferior frontal region (pars triangularis, opercularis and orbitalis), insula, superior, medial and inferior temporal gyri, supramarginal guyrs, angular gyrus, supplementary motor area (SMA) and occipito-temporal area (Benjamin et al. 2017; Labache et al. 2018; Price 2012; Vigneau et al. 2006) with Crus 1 and 2 and IV, V, VI, VII lobules of cerebellum (Keren-Happuch et al. 2014; Price 2012; Stoodley and Schmahmann 2018). In addition, the hippocampus, entorhinal, perirhinal and parahippocampal cortices together with amygdala, cingulum, lateral orbito-frontal gyrus, medial prefrontal cortex, superior and inferior parietal area are specifically involved in encoding and/or retrieval process during long-term memory evaluation (Battaglia et al. 2011; de Vanssay-Maigne et al. 2011; Diana et al. 2007; Ranganath and Ritchey 2012; Spaniol et al. 2009).

In the present study, we present and evaluate an original fMRI protocol entitled GE2REC with the intention to map language-and-memory network in a concise and robust fashion. GE2REC consists of a sentence generation with implicit encoding (GE) in auditory modality and two recollection (2REC) memory tasks, a recognition (RECO) performed in visual modality, and a recall of sentences (RA), performed in auditory modality. The GE and RA runs are designed to activate intermixed language-and-memory network by engaging episodic memory encoding and retrieval respectively, as well as simultaneously, with language processes.

## Material and methods

### Participants

Twenty-one right-handed volunteers aged between 18 and 29 years (M = 21, SD = 3.3; 9 females), without neurological and psychiatric deficits were included in this study. All participants were French native speakers and had normal or corrected-to-normal vision. One participant was excluded from the fMRI analyses due to the high amount of artifacts in the data. This clinical experimentation is governed by the French law (Jardé, Décret n°2016–1537 16/11/2016 from 17/11/ 2016). The Ethic committee for the protection of persons has approved the project (CPP 09-CHUG-14; MS-14-102). All participants provided written informed consent to participate to study and they received financial compensation for their participation.

### Functional MRI (fMRI) assessment of language and memory

**Fig. 1:**
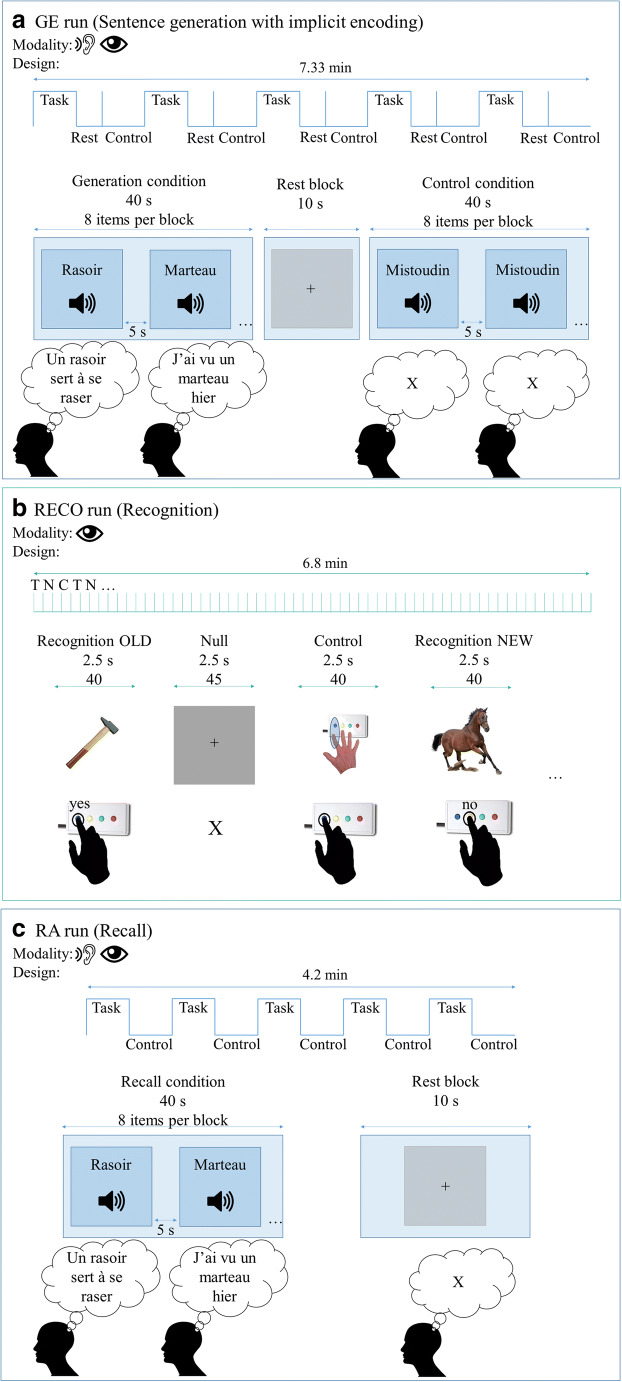
Schematic illustration of the GE2REC protocol. Panel **a:** GE (Sentence generation with implicit encoding) run with block-design. Items were presented in auditory modality during Task (word to generate sentences) and Control (pseudo-word) and in visual modality during Rest (central cross to fixate). Participants were required to covertly generate sentences during Task and to do nothing during Control. They fixated the cross during Rest. Examples of French items are shown *(rasoir = razor; marteau = hammer; mistoudin is a pseudo-word).* Panel **b:** RECO (recognition) run with the event-related design. Items were presented in visual modality during Task (images to recognize), Control (images to be repeated) and Null events (central cross to fixate). Participants were required to recognize whether or not they have heard the object presented in the image and to reply by using the response box. During the Control, they were asked to press the button shown in the picture and to fixate the cross during the Null event. Panel **c:** RA (Recall) run with block-design. Items were presented in auditory modality during Task (word to recall sentences) and in visual modality during Rest (central cross to fixate). Participants were required to recall the sentences they generated in the GE run and to covertly repeat them. They fixated the cross during Rest

The experimental protocol was developed using E-prime software (Psychology Software Tools, Pittsburgh, PA). Before entering into the magnet, the outline of the procedure was explained to participants. Importantly, they only received a full description of the task for the GE run. For the 2REC runs they were only informed about the general outline of the tasks and how they should respond, while they remained uninformed about the actual content of the tasks. A schematic illustration of all tasks is presented in Fig. 1.

#### GE stimuli and task

During the GE run, the participants heard words through a headset and their task was to covertly generate sentences, after hearing a word, that is related to the word they heard and to continue producing the sentences related to this word until they hear the next word. The words have been taken from French standardized naming test D080 (Metz-Lutz et al. 1991). During the GE run participants did not perform the picture naming task, but they produced the sentences in reference to the words they heard. The run included 5 task conditions of sentence generation performed in auditory modality (8 stimuli/condition, 40 words in total) and the inter-stimulus intervals (ISI) that lasted 5 s that were intended to provide enough time to generate a correct sentence. The run also included 5 control periods (non-generation) in order to control for auditory activations during which a pseudoword was played 8 successive times, with 5 s ISI. The participants were asked to listen to the pseudoword and not to talk covertly. The run also included 5 rest blocks with a fixation cross displayed for 10 s, placed directly after the generation blocks in order to provide time for the hemodynamic response to come down. Participants were required to fixate the cross. The order of conditions was Task (Generation), Rest and Control. The total duration of the run was 7.3 min.

#### RECO stimuli and task

During the RECO run performed in the visual modality, the participants were shown pictures on the screen and their task was to respond whether they heard the names of the objects in the images during the GE run. The event-related design was used, including pictures of the words participants heard in the previous task, pictures of the new objects, control images and rest condition. All presented images were real-life equals of the images from the DO80 (Metz-Lutz et al. 1991).^1^ The run included 40 pictures of the words presented in the GE run (henceforth OLD). The participants were instructed to press the “yes” button on their response box that was in their dominant hand when they saw the image that corresponded to one of the words they heard in the previous run. Additionally, the run included 40 pictures of the words that were not presented in the GE run (henceforth NEW). These NEW items (pictures) presented the words that were also taken from the DO80 and these words were matched with the words presented in OLD pictures in terms of lexical length and frequency. The participants were required to press “no” button on their response box when they saw the image that was showing the object whose name they did not hear in the previous run. The run also included 40 control images showing the button that needed to be pressed in order to control for the motor activations during button pressing. Furthermore, the run contained 45 null events represented by a fixation cross. The ISI during RECO task was 2.5 s so all events were displayed during 2.5 s and conditions were presented in pseudo-randomized order. The total duration of the run was 6.8 min. We employed the event-related rather than block design since the former has been shown to identify the effects of successful encoding well (Haag and Bonelli 2013) and in order to avoid the prediction of stimuli. Importantly, there is a change of modality between GE (audio) and RECO tasks (visual) to enhance the access to episodic memory and, accordingly, the activation of hippocampal structures.

#### RA stimuli and task

During the RA run, the participants heard through a headset the words they heard previously in the GE run. Their task was to recall and covertly repeat the sentences they have generated for each word in the GE run and to continue repeating them until hearing the next word. A block design was used, including task and rest conditions. The run included 5 task conditions of recall performed in the auditory modality (8 stimuli/condition, 40 words in total) with 5 s ISI. The run also included 5 rest blocks in visual modality that were represented by a fixation cross displayed for 10 s and participants had to fixate the cross. The total duration of the run was 4.17 min.

Since fMRI is highly sensitive to motion (Powell and Duncan 2005), we have chosen to use covert production in GE and RA runs. This is a commonly used version of production task (Black et al. 2017) that has been proven to provide reliable activation of language regions and lateralization (Benjamin et al. 2017; Haag and Bonelli 2013).

### MR acquisition

Functional MRI was performed at 3T (Achieva 3.0 T TX Philips Medical systems, NL) at IRMaGe MRI facility (Grenoble, France). The manufacturer-provided gradient-echo/T2* weighted EPI method was used for the functional scans. Forty-two adjacent axial slices parallel to the bicommissural plane were acquired in sequential mode (3 mm thickness, TR = 2.5 s, TE = 30 ms, flip angle = 82°, in-plane voxel size = 3 × 3 mm; field of view = 240 × 240 × 126 mm; data matrix = 80 × 80 pixels; reconstruction matrix = 80 × 80 pixels). Additionally, for each participant a T1-weighted high-resolution three-dimensional anatomical volume was acquired, by using a 3D T1TFE (field of view = 256 × 256 × 160 mm; resolution: 1 × 1 × 1 mm; acquisition matrix: 256 × 256 pixels; reconstruction matrix: 256 × 256 pixels).

### Data processing

#### Behavioral analyses

Based on the responses during the RECO run, we calculated behavioral performances during recognition task (%CR_RECO). The encoding performance during GE was indirectly determined via recognition (RECO). On the basis of the %CR_RECO for old items, we identified those that were successfully encoded among all items presented during GE. Statistical analyses were performed using RStudio software version 1.1.456 (RStudio Team 2016). All one-sample and paired *t* tests were computed with “t.test” function in the “stats” R package version 3.5.1 (R Core Team 2018).

#### Functional MRI analyses

The Analyses were performed using SPM12 (Welcome Department of Imaging Neuroscience, London, UK) running under Matlab R2015b (Mathworks Inc., Sherborn, MA, USA).

- Pre-processing steps

Functional MRI volumes were first time-corrected with the mean image as the reference slice to correct artifacts caused by the delay of time acquisition between slices. Thereafter, all time-corrected volumes were realigned to correct the head motion. The T1-weighted anatomical volume was co-registered to mean images obtained through the realignment procedure and normalized to MNI (Montreal Neurological Institute) space. Each normalized functional volume was smoothed by an 8 mm FWHM (Full Width at Half Maximum) Gaussian kernel. Noise and signal drift were removed by using a high-pass filter (1/128 Hz cutoff). Preprocessed data were then statistically analyzed.

- Functional MRI statistical analyses

We evaluated GE and RA runs by analyzing them as a block design, while encoding during sentence generation (ENCO) was analyzed using the GE run but as an event-related design by comparing those GE items that were correctly recognized during the RECO run to those that were not correctly recognized. In the same vein, the recognition was evaluated by analyzing the RECO run as event-related, comparing the correctly recognized items with the ones that were not correctly recognized, as well as comparing correct recognition of OLD and NEW items. Statistical parametric maps were generated from linear contrasts between the HRF parameter estimates for the different experimental conditions. The whole brain effects of interest were firstly evaluated at an individual level (first-level): (1) effect of language by comparing sentence generation and control; (2) effect of memory encoding during sentence generation by comparing the correctly and incorrectly encoded items; (3) effects of memory recognition by comparing correctly with incorrectly recognized items; (4) differences in recognition by comparing recognition of old and new items and (5) effects of memory recall by comparing sentence repetition with the baseline. Six movement parameters obtained by realignment corrections were included as noise (regressors of non-interest).

For the second-level group analysis, individual contrasts were entered into a one-sample *t* test and activations were reported at a *p* < .05 significance level with the FWE corrected (T_GE_ > 6.5; T_ENCO_ > 6.52; T_RECO_ > 7.03; T_RA_ > 6.54) for all effects.

## Results

### Behavioral results

During the RECO run participants correctly recognized on average 72.62% (SD = 10.2) of old items and correctly rejected on average 87.87% (SD = 7.36) of new items. The correct recognition of old items and the correct rejection of new items were both above the chance level (*t*(20)_OLD_ = 10.16, *p* < .001; t(19)_NEW_ = 23.02, *p* < .001). Paired t-test demonstrated that the recognition of old items (M_RT_OLD_ = 0.97; SD = 0.07) was faster (*t*(19) = −5.51, *p* < .001) than the rejection of the new ones (M_RT_OLD_ = 1.1; SD = 0.07).

### Functional MRI

Since the aim of the present paper is to validate the GE2REC protocol in healthy controls we will present the second-level group results. However, the activations obtained for a single participant and the standard deviations across all subjects are provided in the supplementary material (Fig. S1, Table S1) as an illustration of the potential use of this protocol on an individual level.

#### Sentence generation (GE)

**Fig. 2:**
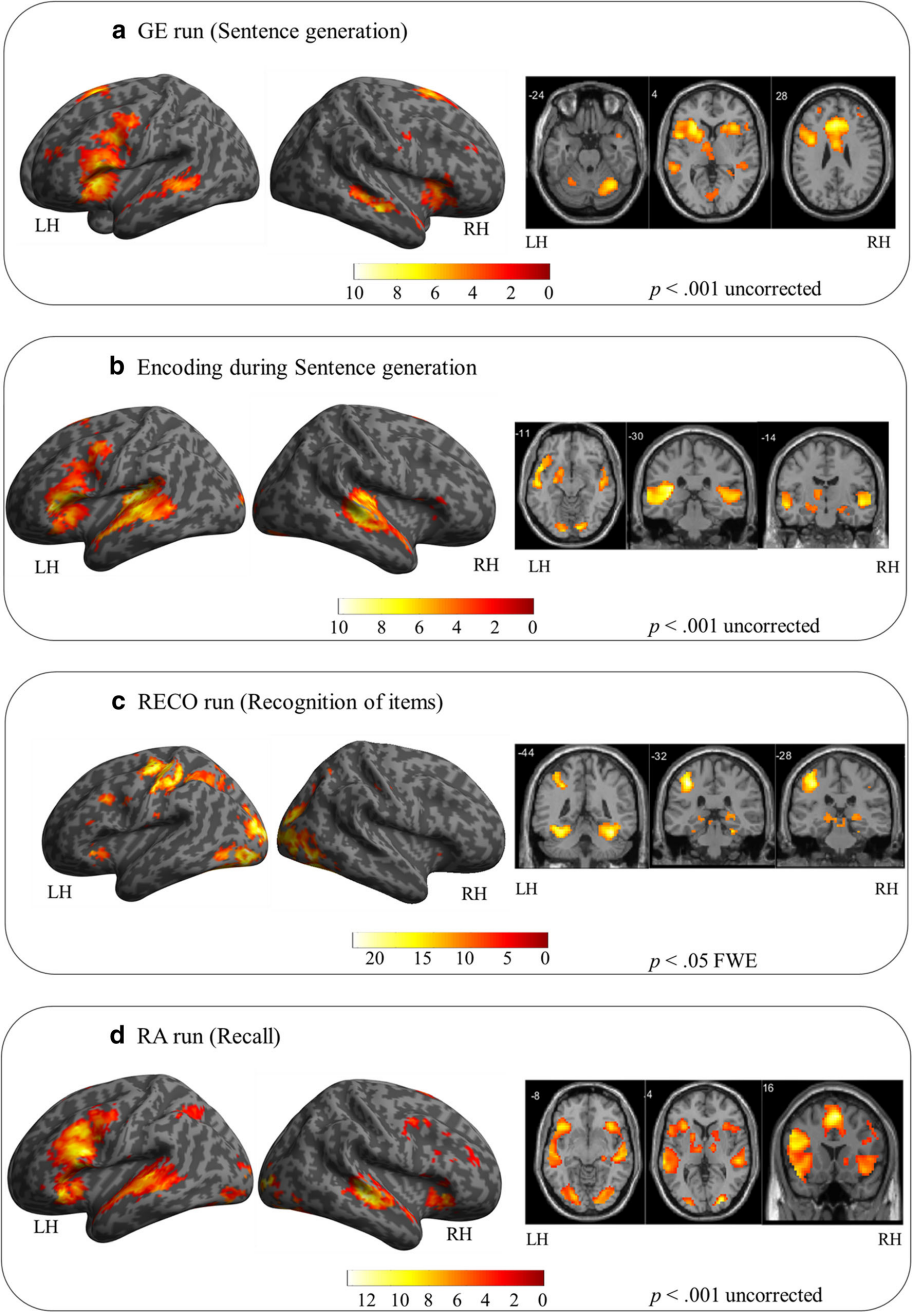
Illustrative overview representation of global activation obtained for sentence generation (panel **a**), encoding (panel **b**), recognition of items (panel **c**) and the recall (panel **d**). Activations for each task were obtained at a group level (*N* = 20 participants for all tasks except recognition of items where *N* = 19 were included due to a lack of responses of one participant). Activations were projected onto the lateral left and right views of surface rendering and 2D coronal and axial slices. The left (LH) and right (RH) hemispheres are indicated. The color scale indicates the T value of the activation. The GE and RA runs, as well as the encoding during sentence generation, were depicted in a more permissive threshold (*p* < .001 uncorrected) in order to illustrate activations that were obtained on this significance level. The presented coronal slices for the encoding during sentence generation were chosen so that they show anterior (y = −14 mm) and posterior (y = −30 mm) hippocampus (Poppenk et al. 2013)

Results of comparing the GE task vs. control are presented in Panel A of Fig. 2 and Table 1. Overall, the results reveal bilateral but predominantly left activation of a large fronto-temporo-parietal network including left prefrontal, inferior frontal, bilateral insula and right precuneus. The activation of left superior temporal and bilateral middle temporal and superior temporal pole cortices were also observed together with right cerebellum Crus 1 and VI.

**Table 1:** Activated regions for the contrast GE vs. Control. The number of voxels in the cluster (k), the x, y and z coordinates in millimetres, the anatomical region according to AAL atlas (Tzourio-Mazoyer et al. 2002), the Brodmann Area (BA) and the T value are indicated for each peak. All activations were obtained at *p* < .05 corrected except for those with asterisks in the table (*p < .001 uncorrected). Abbreviation: GE = sentence Generation

Contrast	k	x (mm)	y (mm)	z (mm)	AAL	BA	T
GE [Sentence generation vs. Control]	383	-3	14	50	Supp_Motor_Area_L	6	10.21
		3	14	50	Supp_Motor_Area_R	6	8.88
		−6	23	32	Cingulum_Mid_L		9.85
		15	20	35	Cingulum_Mid_R		7.21
		−9	17	44	Frontal_Sup_Medial_L		8.81
		−6	26	29	Cingulum_Ant_L		8
		12	26	23	Cingulum_Ant_R		7.1
		6	20	44	Frontal_Sup_Medial_R	32	7.45
		−12	14	47	Frontal_Sup_L		6.93
		−39	20	−7	Frontal_Inf_Orb_L		6.7*
		−51	8	−4	Temporal_Pole_Sup_L	38	4.64*
	11	6	11	23	Cingulate_Ant_R		8.48
	16	48	−25	−7	Temporal_Mid_R		8.17
	37	39	−55	−31	Cerebelum_Crus1_R		8.14
		33	−55	−31	Cerebelum_6_R		7.36
	43	−24	23	2	Insula_L		8.1
	15	−54	20	17	Frontal_Inf_Tri_L	45	7.55
		−54	11	11	Frontal_Inf_Oper_L	44	6.59
	17	6	−70	−16	Vermis_6		7.14
	239	−51	−34	−1	Temporal_Mid_L	21/22	6.07*
		−48	−34	5	Temporal_Sup_L		3.59*
	31	30	−49	8	Precuneus_R		4.44*

*At *p* < .001 uncorrected

#### Encoding during the sentence generation (ENCO)

Correct encoding of the items during generation of sentences activated expected language regions such as left inferior frontal and bilateral middle and superior temporal cortices. Bilateral hippocampal activation was also detected with lower significance level (*p* < .001). These activations are presented in the Panel B of the Fig. 2 and Table 2.

**Table 2:** Activated regions for the Encoding during sentence generation (GE task) obtained as a contrast between items that have later been correctly or incorrectly recognized (RECO task), modelled as an event-paradigm. The number of voxels in the cluster (k), the x, y and z coordinates in millimetres, the anatomical region according to AAL atlas (Tzourio-Mazoyer et al., 2002), the BA and the T value are indicated for each peak. All activations were obtained at *p* < .05 corrected except for those with asterisks in the table (*p < .001 uncorrected). Abbreviation: ENCO = Encoding during sentence generation

Contrast	k	x (mm)	y (mm)	z (mm)	AAL	BA	T
ENCO [correct vs. incorrect]	101	−51	20	−4	Frontal_Inf_Orb_2_L		10.20
		−48	14	14	Frontal_Inf_Oper_L		9.19
		−48	29	2	Frontal_Inf_Tri_L		8.81
	161	−42	−37	**20**	Temporal_Sup_L		9.84
		−63	−28	2	Temporal_Mid_L		7.42
		−18	−13	−16	Hippocampus_L		4.27*
		−51	8	−10	Temporal_Pole_Sup_L		6.56
	71	−3	5	68	Supp_Motor_Area_L		9.23
	105	39	−25	5	Heschl_R		8.56
		51	−25	2	Temporal_Sup_R		7.5
		51	−22	−7	Temporal_Mid_R		6.72
	24	33	−10	−22	Hippocampus_R		4.56*
		21	−13	−19	ParaHippocampal_R		4.06

*At *p* < .001 uncorrected

#### Recognition (RECO)

Correct retrieval process during the recognition task (task vs. control) activated a large frontal-temporo-parietal network shown in Panel C of Fig. 2 and Table 3. The identified network included bilateral fusiform gyri and occipital cortices, left inferior and superior parietal cortices, left cingulum, medial prefrontal cortex, left inferior and orbito-frontal gyrus, left insula and bilateral hippocampi. Bilateral parahippocampal activation was also detected with lower significance level (*p* < .001). Correct recognition activated also bilateral cerebellum IV-V and VI as well as left lobe Crus 1.

**Table 3:** Activated regions for the contrast Correct vs. Incorrect during the RECO task. The number of voxels in the cluster (k), the x, y and z coordinates in millimetres, the anatomical region according to AAL atlas (Tzourio-Mazoyer et al., 2002), the BA and the T value are indicated for each peak. All activations were obtained at p < .05 corrected except for those with asterisks in the table (*p < .001 uncorrected). Abbreviation: RECO = recognition of items

Contrast	k	x (mm)	y (mm)	z (mm)	AAL	BA	T
RECO [Correct vs. Incorrect]	3356	−36	−82	−4	Occipital_Mid_L		17.07
		18	−94	11	Cuneus_R		16.75
		42	−73	−10	Occipital_Inf_R		16.29
		−30	−52	−13	Fusiform_L		14.21
		36	−55	−16	Fusiform_R		12.5
		−42	−46	−16	Temporal_Inf_L	37	12.26
		−30	−61	−22	Cerebellum_6_L		12.17
		15	−85	−10	Lingual_R		11.7
		−24	−64	−10	Lingual_L		11.32
		48	−52	−16	Temporal_Inf_R	37	11.17
		27	−58	−22	Cerebellum_6_R		10.84
		−24	−46	−22	Cerebellum_4_5_L		10.69
		24	−46	−22	Cerebellum_4_5_R		9.77
		−42	−58	−4	Temporal_Mid_L		9.26
		−42	−67	−22	Cerebellum_Crus_1_L		8.41
		36	−22	−22	ParaHippocampal_R		5.25*
		−27	−28	−22	ParaHippocampal_L		6.05*
	788	−39	−31	50	Postcentral_L		16.99
		−39	−19	56	Precentral_L	3	15.31
		−39	−40	50	Parietal_Inf_L		10.67
		−24	−67	47	Parietal_Sup_L	7	10.67
		−54	−22	41	SupraMarginal_L		9.35
	154	−3	−4	53	Supp_Motor_Area_L		**10.87**
		0	23	44	Frontal_Sup_Medial_L		8.56
		0	−4	50	Cingulum_Mid_L		8.34
		3	23	41	Frontal_Sup_Medial_R		7.5
	92	−45	11	32	Precentral_L		11.4
		−48	14	32	Frontal_Inf_Oper_L		10.05
		−42	8	35	Frontal_Mid_L		8.88
		−42	14	26	Frontal_Inf_Tri_L		7.35
	61	−36	26	−1	Frontal_Inf_Tri_L		11.4
		−30	26	−1	Insula_L		10.13
		−36	26	−4	Frontal_Inf_Orb_L		9.97
	57	−15	−25	−1	Thalamus_L		8.47
		−15	−31	−4	Hippocampus_L		7.77
	29	24	−31	2	Hippocampus_R		8.79
	17	−57	−16	20	Postcentral_L		8.56
	15	0	2	26	Cingulate_Ant_L		9.47

*At *p* < .001 uncorrected

#### Differences in recognition

The comparison of two types of items showed that the recognition of old items engaged more the left parietal cortex, notably precuneus, cuneus and angular gyrus, as well as bilateral middle cingulate and middle temporal cortices. Conversely, correctly rejecting new items in comparison to correctly recognizing old ones activated more bilateral fusiform and occipital regions. The activations are presented in the Table 4.

**Table 4:** Activated regions for the contrast RECO_OLD vs. RECO_NEW and the opposite contrast. The number of voxels in the cluster (k), the x, y and z coordinates in millimetres, the anatomical region according to AAL atlas (Tzourio-Mazoyer et al., 2002), the BA and the T value are indicated for each peak. All activations were obtained at p < .05 corrected except for those with asterisks in the table (*p < .001 uncorrected). Abbreviation: RECO_OLD = recognition of OLD items; RECO_NEW = recognition of NEW items

Contrast	k	x (mm)	y (mm)	z (mm)	AAL	BA	T
RECO_OLD vs. RECO_NEW	210	−6	−67	35	Precuneus_L	7	10.59
		−6	−64	26	Cuneus_L	31	9.91
	31	−45	−61	41	Angular_L		8.47
	30	3	−25	35	Cingulate_Mid_R	24	8.10
		−6	−43	35	Cingulate_Mid_L	31	7.30
	8	−45	−52	14	Temporal_Mid_L		7.91
	5	60	−49	8	Temporal_Mid_R		7.89
RECO_NEW vs. RECO_OLD	168	27	−67	−7	Fusiform_R		6.47*
		27	−52	−10	Lingual_R		4.33*
	143	−27	−85	−10	Occipital_Inf_L		6.19*
		−24	−88	−1	Occipital_Mid_L		5.48*
		−27	−70	−7	Fusiform_L		4.87*
	21	−27	−49	−16	Occipital_Mid_L		4.77*
	11	−33	−82	20	Occipital_Mid_R		4.69*

*At *p* < .001 uncorrected

#### Recall (RA)

The recall process (recall vs. baseline) activated a network presented in Fig. 2, Panel D and Table 5 that consisted of left inferior frontal and bilateral predominantly right oriented prefrontal and medial frontal cortices and left insula. Bilateral activations in temporal superior and middle cortices as well as left temporal pole were also identified. The activation of the parietal regions consisted of the left inferior parietal and angular gyrus, while the activations of the cerebellum were limited to right Crus 1. Right hippocampal activation was also detected with lower significance level (*p* < .001).

**Table 5:** Activated regions for the contrast RA vs. baseline. The number of voxels in the cluster (k), the x, y and z coordinates in millimetres, the anatomical region according to AAL atlas (Tzourio-Mazoyer et al., 2002), the BA and the T value are indicated for each peak. All activations were obtained at *p* < .05 corrected except for those with asterisks in the table (*p < .001 uncorrected). Abbreviation: RA = recall

Contrast	k	x (mm)	y (mm)	z (mm)	AAL	BA	T
RA [Recall vs. Baseline]	285	−45	8	23	Frontal_Inf_Oper_L		13.21
		−45	18	17	Frontal_Inf_Tri_L		8.74
		−36	5	29	Precentral_L		6.89
		−41	8	35	Frontal_Mid_L		7.52
		−45	−37	14	Temporal Sup L		5.69*
		−42	14	−19	Temporal Pole Sup L	38	5.55*
	224	3	17	53	Supp_Motor_Area_R	8	11.61
		−3	17	50	Supp_Motor_Area_L	8	10.32
		0	23	44	Frontal_Sup_Medial_L		10.74
		3	23	44	Frontal_Sup_Medial_R		10.44
		9	26	38	Cingulum_Mid_R	6	7.24
		−12	20	44	Frontal_Sup_L		5.17*
	118	54	−28	2	Temporal_Sup_R		10.96
		48	−22	−7	Temporal Mid R		6.74
	71	27	−88	5	Occipital_Mid_R		10.72
		27	−82	−16	Fusiform_R		7.31
		27	−88	5	Occipital_Mid_R		10.72
		30	−88	−5	Occipital Inf R	18	7.02
		15	−91	−7	Lingual R		6.86
		24	−25	−7	Hippocampus_R		4.87*
	45	36	26	−1	Insula R		9.09
		33	26	−7	Frontal_Inf_Orb_R		7.52
	108	−33	23	2	Insula L		9.08
		−42	26	−4	Frontal_Inf_Orb_L		9.07
	92	−63	-31	2	Temporal Mid L	22	8.82
		−63	−19	5	Temporal_Sup_L		6.68
	68	-18	−94	−1	Occipital Mid L		8.81
		−21	−85	−13	Lingual L		8.25
		−21	−82	−7	Fusiform L		8.01
		−21	−88	−4	Occipital_Inf_L		7.08
	16	9	−73	−28	Cerebelum_Crus1_R		8.24
	10	−33	−52	38	Parietal_Inf_L		8.21
		−33	−52	35	Angular_L		7.69*

*At *p* < .001 uncorrected

Although the RA task was designed to explore the interaction of language and memory, in order to check if this task indeed engaged memory in addition to language processes, a paired t-test was conducted testing for activation differences between RA and GE task. This analysis indicated that the RA task engaged more bilateral lateral and medial parietal regions as well as the right hippocampus when employing a lower significance level (*p* < .001) as shown in Table 6.

**Table 6:** Activated regions for the paired t-test RA vs. GE. The number of voxels in the cluster (k), the x, y and z coordinates in millimetres, the anatomical region according to AAL atlas (Tzourio-Mazoyer et al., 2002), the BA and the T value are indicated for each peak. All activations were obtained at *p* < .05 corrected except for those with asterisks in the table (*p < .001 uncorrected). Abbreviation: RA = recall; GE = sentence Generation

Contrast	k	x (mm)	y (mm)	z (mm)	AAL	BA	T
RA – GE [Recall vs. Sentence generation]	21	−6	−67	32	Precuneus_L	7	7.98
		3	−64	32	Precuneus_R	7	6.25*
		−6	−73	32	Cuneus_L		5.28*
		3	−76	35	Cuneus_R		3.64*
	15	−27	−79	−19	Fusiform_L		6.93
	25	27	−25	−7	Hippocampus_R		3.93*
		−33	−55	38	Parietal_Inf_L		6.12*
		−36	−55	38	Angular_L	40	5.32*
	47	36	−70	44	Angular_R		4.85*

*At *p* < .001 uncorrected

**Fig. 3:**
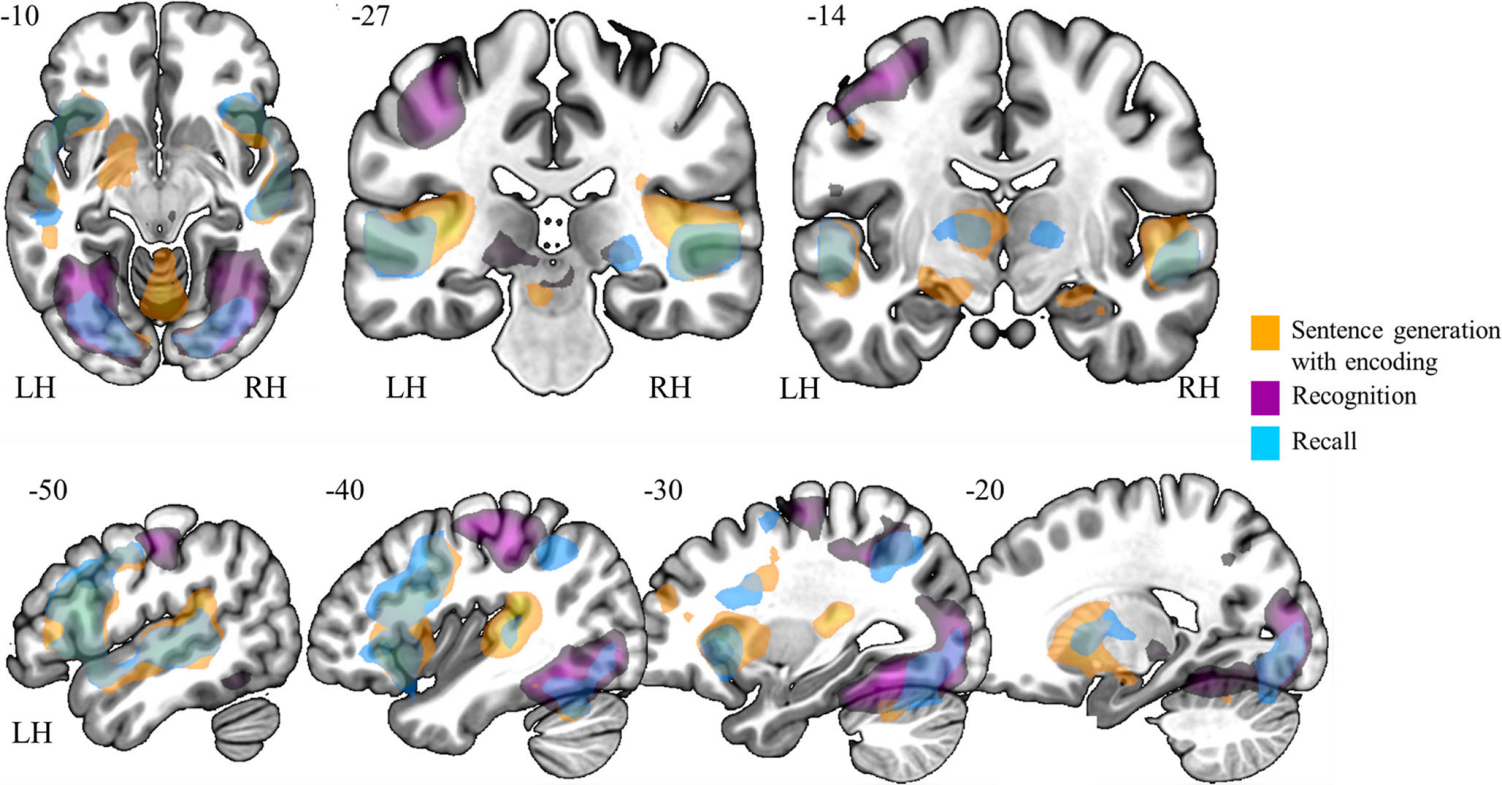
Illustrative overview of the synthesis of results obtained with GE2REC protocol during sentence generation with encoding (orange), recognition of items (violet) as well as the recall (blue). The activated regions are projected onto 2D anatomical slices presented in axial, coronal and sagittal orientations. The left (LH) and right (RH) hemispheres are indicated

Figure 3 presents the synthesis of the results. The principal findings can be summed up as follows: (a) sentence generation activated bilateral temporal, left frontal and parietal regions, (b) implicit encoding of the items into the long-term memory during sentence generation engaged bilateral hippocampi in addition to language regions (c) correct recognition of the items activated bilateral inferior ocipito-temporal, left parietal and bilateral hippocampal and parahippocampal regions, but also the left frontal inferior, SMA and (d) recall activated large fronto-temporo-parietal network with the right hippocampus.

## Discussion

The interaction between language and memory plays an essential role in our everyday lives, the most obvious example being that it allows us to hold meaningful conversations with memory providing the basis for tracking and maintaining proper conversational flow. To explore this interaction, we need an adequate tool that would be able to capture this synergy in action while being adapted to both clinical settings and empirical research standards. To this end, we propose the GE2REC protocol developed for interactive mapping of the language-and-memory network and present its validation in healthy participants.

Our results indicate that sentence generation activated a large bilateral, but predominantly left fronto-temporo-parietal network. Despite the covert production, this network included left inferior frontal (pars opercularis and pars triangularis), left insula and bilateral SMA usually required by the production of sentences (Grande et al. 2012; Haller et al. 2005; Menenti et al. 2012; Price 2012; Segaert et al. 2012), while the inhibition of articulation could explain the activation of anterior cingulum (Lœvenbruck et al. 2018; Price 2012). Additionally, superior and middle temporal gyri as well as the superior temporal pole were activated, which is in line with other results reporting syntactic, lexical-semantic and phonological demands during a sentence generation (Grande et al. 2012; Indefrey and Levelt 2004; Menenti et al. 2012; Price 2012; Segaert et al. 2012). Nevertheless, apart from the right precuneus, the GE task did not elicit activations in standard language parietal regions (Price 2012) which could be due to the fact that this task did put too much demand on phonological processing (like rhyming tasks do) and speech comprehension (Cousin et al. 2007). The successful encoding during sentence generation showed the bilateral hippocampal activation on a more permissive threshold (*p* < .001) which is in line with previously reported findings (Diana et al. 2007; Preston and Eichenbaum 2013; Spaniol et al. 2009), even though the expected prefrontal activation was not observed. Also, the obtained hippocampal activation tended to be rostral (anterior), in line with previous studies and models (Lepage et al. 1998; Preston and Eichenbaum 2013; Spaniol et al. 2009). Employment of the permissive threshold can be justified having in mind that fMRI acquisition of medial temporal lobe can be affected by geometric distortions and signal loss (Haag and Bonelli 2013; Powell and Duncan 2005).

The change in the modality between GE (auditory) and RECO (visual) run was implemented in this protocol with the intention of eliciting participants’ responses based on recognition, rather than familiarity, activating thus episodic memory (Perrone-Bertolotti et al. 2015). We believe our participants really did remember instead of relying on familiarity of the stimuli since the network activated by RECO corresponded well with the “Binding of Item and Context” model (Diana et al. 2007) and the episodic posterior medial network proposed by Ranganath and Ritchey (2012) in that it indeed activated posterior bilateral hippocampal and parahippocampal gyri, as well as cingulate, lateral parietal and prefrontal cortices. Although we obtained bilateral instead of right prefrontal activation predicted by the HERA model (Habib et al. 2003), our results are in agreement with previous findings (Spaniol et al. 2009). Additionally, we found expected (Guerin and Miller 2009) differences between correctly identified old items and correctly rejected new items reflected in reaction time and left parietal activation. Although previous studies connected the activation of fusiform gyrus, inferior frontal cortex and insula with encoding and retrieval processes (Aldenkamp et al. 2003; Spaniol et al. 2009), we believe that the activations of these regions we identified during recognition may reflect a verbal strategy used by participants to perform the task which included picture naming. Activations found in inferior frontal, SMA, insula, fusiform and parietal cortices indeed correspond well with the picture naming network (Duffau et al. 2014). Additionally, identified cerebellar activations, specifically Crus 1 and lobules IV-V and VI, correspond to language processes (Keren-Happuch et al. 2014; Price 2012; Stoodley and Schmahmann 2018). These results suggest that trying to separate language and memory functions is probably artificial and they should instead be assessed realistically in a dynamic interaction, especially when it comes to patients. Having in mind, for instance, that TLE is often accompanied by HS with implications on both language and memory (Alessio et al. 2006; Bonelli et al. 2011; Davies et al. 1998; Zalonis et al. 2017), it is crucial that the protocol used in preoperative mapping has the ability to robustly activate hippocampal and neighbouring structures. We have seen that the GE2REC protocol can activate these structures both during encoding and recognition memory processes.

Finally, the RA task was designed to directly assess the interactive dynamics of language-and-memory while also being close to everyday experiences by having a more natural recollection context. The RA activations of the left inferior frontal gyrus, bilateral SMA and insula as well as bilateral superior and middle temporal cortices and Crus 1 of the cerebellum resembled the ones found during generation and can be related to the language component of the network (Hickok and Poeppel 2007; Indefrey and Levelt 2004; Price 2012). On the other hand, the activations of the bilateral prefrontal and predominantly left parietal cortices as well as bilateral fusiform gyri, are in agreement with the previous results on memory retrieval (Aldenkamp et al. 2003; Spaniol et al. 2009). It should also be noted that some structures that were active during this task have previously been found to be active both in language and memory tasks. For example, temporo-polar cortex, lateral orbitofrontal and angular gyrus make up a part of the two memory systems (Ranganath and Ritchey 2012), while at the same time being involved in language networks and engaged in semantic processing (Duffau et al. 2014; Price 2012). Additionally, the occipito-temporal, parietal and hippocampal RA activations match the subsystem that was suggested to represent the link between inner representations and episodic memory (Vandenberghe et al. 2013). This again supports the idea of a large language-and-memory network and shows that these regions are activated when the individual is engaged in mixed language-and-memory tasks and situations. The supplementary analysis comparing RA and GE tasks further supports that the RA task did not rely exclusively on language processes and that it was indeed based on both processes engaging parts of memory network such as parietal cortices and hippocampal structures. Even though left activation of the hippocampus is expected during this task due to the verbal nature of the material (Witt et al. 2019), we observed right activation of this structure during the RA task. One potential explanation could be the fact that participants performed visual RECO task just before doing the RA task. Namely, participants could have linked the images of the words they saw in the RECO task with the sentences they have generated during the GE task with the reference to the same words. Therefore, during the RA task they did not retrieve just the phrases they produced during the first task, but they recalled integrated vivid episodes that also included the images seen in the second task. Due to this, their episodes had a strong visual aspect. This would explain the activation of the right hippocampus that has been found to be engaged in the perceptual episodic memory (St-Laurent et al. 2016). This could also reflect the strategy of relying largely on visual aspects of the episode during the recall.

Importantly, although the hippocampus was proposed to be included in the language network (Covington and Duff 2016), we observed its activation only during the sentence task (GE) when focusing on the difference between correctly and incorrectly encoded items. Nevertheless, this does not refute the implication of the hippocampus in language processes since there are several explanations for the lack of activations. First of all, it could be that the hippocampus is implied in other aspects of language processing that we have not included in the GE task such as sentence comprehension (Piai et al. 2016; Pu et al. 2020), while it was active during picture naming that we assume was performed during RECO task. Secondly, it was proposed that comprehension of familiar words (such as those used in our protocol) activate nodes that have already formed connections, so there is no need for new connection formation and hippocampal activity (MacKay et al. 1998). Finally, our results could also suggest that the hippocampus is perhaps not a primary element of the exclusive language network, but that it is instead a part of the language-and-memory network, connecting the two systems.

Overall, the wide additive network (Fig. 3) recruited by the GE2REC protocol, can be considered as the interactive language-and-memory network since it was obtained through the linked tasks in which two processes were highly intertwined. It is also important to note that this cerebral substrate of combined and intermixed language and memory processes has specific anatomical support. Specifically, the mesial temporal, temporal pole and prefrontal cortices could be inter-connected via the direct inter-hippocampal pathway, while the polysynaptic pathway could connect parietal and temporal cortices through the parahippocampal gyrus towards cingulate cortices (Duvernoy et al. 2013). Additionally, anterior temporal and orbito-frontal areas that have been found during RA could be connected via UF that supports both functions (Diehl et al. 2008; Duffau et al. 2014; McDonald et al. 2008). IFOF could connect frontal and occipital regions, supporting semantic processing, verbal memory and noetic consciousness (McDonald et al. 2008; Moritz-Gasser et al. 2013). Nevertheless, one of the next steps of this line of research will be to explore structural and functional connectivity within the GE2REC language-and-memory network.

We believe that by combining language and memory, the GE2REC protocol may have important clinical implications. First, it allows mapping language and memory networks as well as their joint cooperative network during a short scan. Secondly, previous studies on mapping the neural overlap between cognitive processes pointed out that group level activations are not necessarily found on individual level (Fedorenko et al. 2013), especially in the case of mesial temporal structures (Saddiki et al. 2018). This protocol increases the access to mesial temporal structures, crucial for preoperative planning, by encompassing two recollecting memory tasks in different modalities. Unravelling the complex interaction between two cognitive functions is important from a clinical perspective, for (i) furthering our understanding of how each function potentially contributes to a specific cognitive deficit, (ii) allowing for greater accuracy and precision when predicting cognitive deficits resulting from brain lesions or following surgery, and (iii) developing more interactive neuro-rehabilitation tools based on this interaction to indirectly improve a given function (language, for instance) by reinforcing the function it interacts with (such as memory).

### Limitations

Our work has several limitations, the first being that due to covert speech, participants’ responses for the GE and RA tasks cannot be recorded and performance on these tasks cannot be measured. Nevertheless, as previous studies employing the covert instead of overt response modality (Benjamin et al. 2017; Haag and Bonelli 2013), we also identified expected cognitive networks. Secondly, although above the chance level, participants’ responses during RECO were not as highly accurate as expected (Marcela Perrone-Bertolotti et al. 2015). The reason for this could be that participants were not explicitly instructed to memorize the items they heard during GE. Also, drawing on the important capacity of episodic memory to flexibly retrieve and recombine information from distinct past experiences (Carpenter and Schacter 2017) and given that the images used during RECO are frequently encountered in the everyday life, participants could have mistakenly combined features of different episodes. Although it would be very informative to test the language and memory interaction in cases when one of the two is severely damaged, the usage of this protocol demands a certain level of function perseveration which limits its application for some pathologies. For instance, GE2REC would have limited application in patients with Alzheimer’s disease (Montembeault et al. 2019) or severe cases of aphasia. Finally, GE2REC protocol should mainly be used for patients with anterior-temporal and frontal EZ and should be used with precaution for patients with EZ or lesion in parietal regions especially supramarginal girus, seeing as we did not identify the activation in this region. Nevertheless, GE2REC protocol does not aim towards general and exhaustive assessment of language and memory because many linguistic and memory aspects are not explored by GE2REC and it was designed with the intention to be used mainly with TLE patients.

### Conclusion

In this study, we proposed and validated the GE2REC protocol for interactive mapping of a global language-and-memory network with healthy patients. GE2REC is easy to perform, has short duration and sufficiently robust activation. Furthermore, it can jointly activate a large fronto-temporo-parietal network generally observed in language studies, as well as mesial temporal, parietal and prefrontal cortices, generally reported by memory studies. In addition, with respect to memory, it explores both encoding and retrieval processes and allows for left-right and anterior-posterior segregation of their cerebral representations. By synthesizing the results of its three tasks designed to explore the interactive nature of language-and-memory, GE2REC provides the cartography of this network which could be of practical importance.

## Electronic supplementary material

ESM 1 (DOCX 1.08 mb)

## Footnotes

1 We chose to use different, real images rather than original ones from DO80 test, for two reasons. First, the original images are black-and-white drawings and taking the real images of objects was closer to everyday experience. And more importantly, DO80 is frequently used in behavioral experiments and neuropsychological evaluation of patients in our environment. Thus, we wanted to avoid the recognition of items based on familiarity.
