# Correction to: Interactive mapping of language and memory with the GE2REC protocol

**DOI:** 10.1007/s11682-020-00403-6

**Published:** 2020-10-01

**Authors:** Sonja Banjac, Elise Roger, Emilie Cousin, Marcela Perrone-Bertolotti, Célise Haldin, Cédric Pichat, Laurent Lamalle, Lorella Minotti, Philippe Kahane, Monica Baciu

**Affiliations:** 1 University of Grenoble Alpes, CNRS LPNC UMR 5105, F-38000 Grenoble, France; 2 University of Grenoble Alpes, UMS IRMaGe CHU Grenoble, F-38000 Grenoble, France; 3 University of Grenoble Alpes, GIN, Synchronisation et modulation des Réseaux Neuronaux dans l’Epilepsie’ and Neurology Department, F-38000 Grenoble, France

**Correction to: Brain Imaging and Behavior.**

10.1007/s11682-020-00355-x

The article Interactive mapping of language and memory with the GE2REC protocol, written by Sonja Banjac, Elise Roger, Emilie Cousin, Marcela Perrone-Bertolotti, Célise Haldin, Cédric Pichat, Laurent Lamalle, Lorella Minotti, Philippe Kahane, and Monica Baciu was originally published electronically on the publisher’s internet portal on August 06, 2020 without open access. With the author(s)’ decision to opt for Open Choice the copyright of the article changed on September 10, 2020 to © The Authors 2020 and the article is forthwith distributed under a Creative Commons Attribution 4.0 International License, which permits use, sharing, adaptation, distribution and reproduction in any medium or format, as long as you give appropriate credit to the original author(s) and the source, provide a link to the Creative Commons licence, and indicate if changes were made.

The images or other third party material in this article are included in the article’s Creative Commons licence, unless indicated otherwise in a credit line to the material. If material is not included in the article’s Creative Commons licence and your intended use is not permitted by statutory regulation or exceeds the permitted use, you will need to obtain permission directly from the copyright holder.

To view a copy of this licence, visit http://creativecommons.org/licenses/by/4.0/.

