# Supplementary material for: Interactive mapping of language and memory with the GE2REC protocol
Source: Brain Imaging Behav. 2020 Aug 6;15(3):1562–79. doi: 10.1007/s11682-020-00355-x (PMC8286228; doi:10.1007/s11682-020-00355-x)
Supplement: Supplementary file 1 — (DOCX 1.08 mb) [file 11682_2020_355_MOESM1_ESM.docx]

**
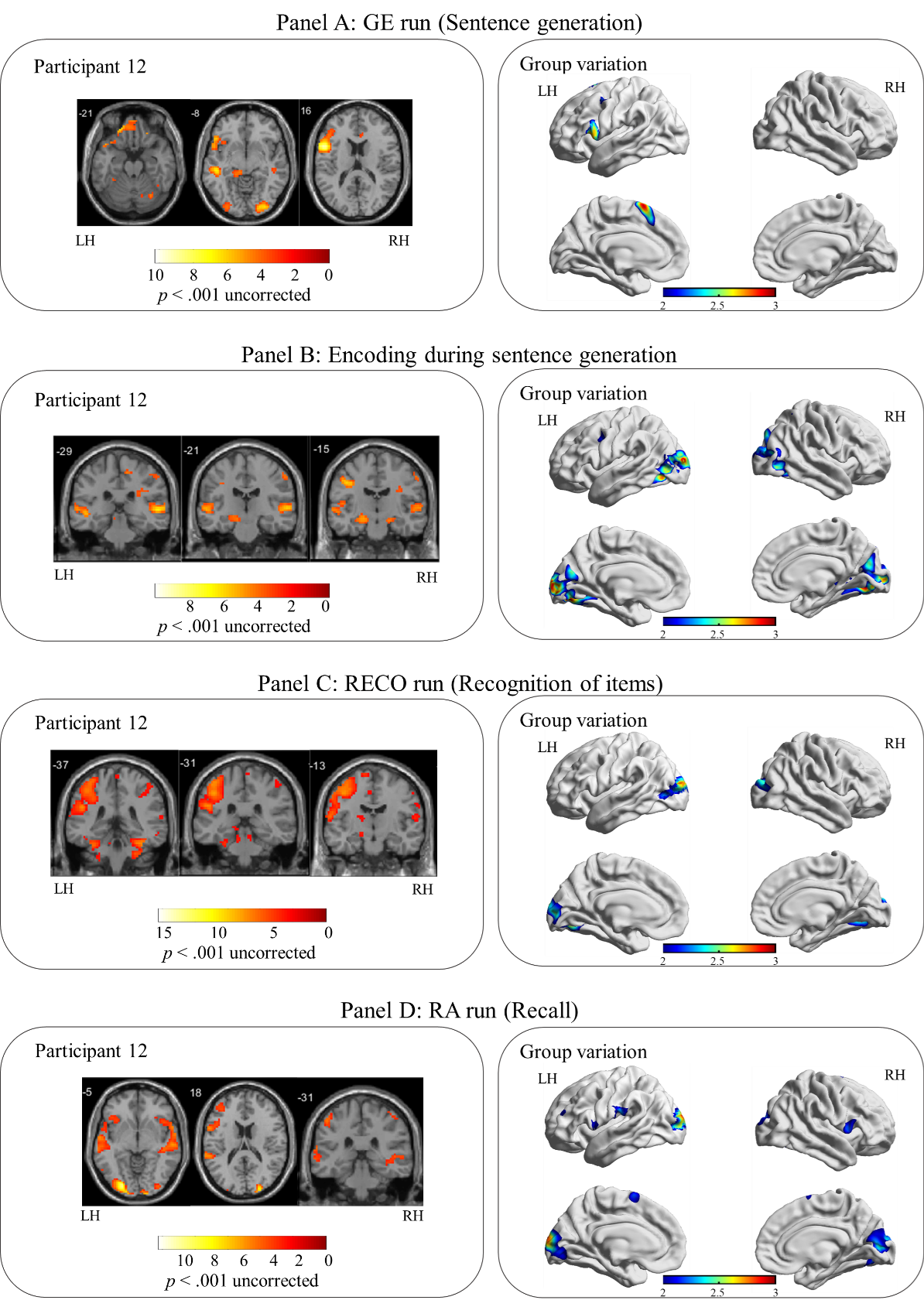
Figure S1**. Illustration of the possibility of application of GE2REC protocol on the individual level. On the left we present an example of activations obtained in one participant and on the right standard deviations across all subjects for sentence generation (panel A), encoding during sentence generation (panel B), recognition of items (panel C) and the recall (panel D). The computation of standard deviations was performed based on individual contrasts mapped on the brain surface and by using the "std" Surfstat function ([https://www.nitrc.org/projects/surfstat)](https://www.nitrc.org/projects/surfstat) running on Matlab R2019a (Mathworks Inc., Sherborn, MA, USA). The most variable areas (> 2SD) from one individual to another are shown on a 3D brain render, from blue to red using BrainNet (Xia, Wang & He, 2013).

| **Contrast** | **k** | **x (mm)** | **y (mm)** | **z (mm)** | **AAL** | **BA** | **T** |
| --- | --- | --- | --- | --- | --- | --- | --- |
| GE [Sentence generation vs. Control] | 198 | -54 | 11 | 20 | Frontal_Inf_Oper_L | 45 | 10.18 |
|  |  | -54 | 17 | 23 | Frontal_Inf_Tri_L |  | 7.87 |
|  |  | -42 | 38 | 29 | Frontal_Mid_2_L |  | 5.08 |
|  | 288 | -3 | 11 | 53 | Supp_Motor_Area_L |  | 9.59 |
|  |  | -3 | 23 | 38 | FrontaL_Sup_Medial_L |  | 6.63 |
|  |  | -12 | 17 | 47 | Frontal_Sup _L |  | 5.89 |
|  |  | -6 | 23 | 35 | Cingulum_Mid_L | 32 | 5.83 |
|  | 135 | -48 | -1 | 47 | Precentral_L |  | 9.31 |
|  |  | -51 | -7 | 41 | Postcentral_L |  | 6.91 |
|  | 91 | -42 | 20 | -19 | Temporal_Pole_Sup_L |  | 7.07 |
|  |  | -48 | 26 | -7 | Frontal_Inf_Orb_2_L |  | 5.68 |
|  | 79 | -48 | -28 | -4 | Temporal_Mid_L |  | 7.00 |
|  | 21 | -60 | -58 | 2 | Temporal_Mid_L |  | 6.99 |
|  | 29 | -21 | 41 | -19 | Frontal_Mid_Orb_L | 11 | 6.86 |
|  | 41 | -33 | 17 | 8 | Insula_L |  | 6.83 |
|  |  | -39 | 29 | 2 | Frontal_Inf_Tri_L |  | 4.85 |
|  | 83 | 30 | -88 | -4 | Occipital_Inf_R | 18 | 6.65 |
|  |  | 24 | -88 | -4 | Lingual_R |  | 6.41 |
|  | 13 | 45 | 38 | 32 | Frontal_Mid_2_R |  | 5.97 |
|  | 22 | -45 | -37 | 53 | Parietal_Inf_L | 40 | 5.73 |
|  | 12 | 42 | -61 | -34 | Cerebelum_Crus1_R |  | 5.12 |
| ENCO [Correct vs. Incorrect] | 216 | -48 | -7 | 44 | Precentral_L |  | 9.48 |
|  | 183 | -54 | 5 | 23 | Postcentral_L |  | 8.23 |
|  |  | -51 | 29 | 23 | Frontal_Inf_Tri_L | 46 | 5.72 |
|  |  | -54 | 14 | 20 | Frontal_Inf_Oper_L |  | 7.62 |
|  | 190 | -3 | 11 | 53 | Supp_Motor_Area_L | 6 | 6.87 |
|  |  | -15 | 17 | 47 | Frontal_Sup_L |  | 5.63 |
|  | 52 | 57 | -28 | 2 | Temporal_Sup_R | 22 | 6.19 |
|  | 248 | 30 | -61 | -25 | Cerebelum_6_R |  | 6.06 |
|  |  | 9 | -88 | -7 | Lingual_R |  | 5.89 |
|  |  | 36 | -67 | -31 | Cerebelum_Crus1_R |  | 5.01 |
|  | 11 | -57 | -58 | 2 | Temporal_Mid_L |  | 6.00 |
|  | 75 | 15 | -79 | -49 | Cerebelum_7b_R |  | 5.91 |
|  |  | 12 | -67 | -52 | Cerebelum_8_R |  | 4.83 |
|  |  | 15 | -79 | -43 | Cerebelum_Crus2_R |  | 5.31 |
|  | 77 | -57 | -40 | 20 | Temporal_Sup_L |  | 5.88 |
|  |  | -48 | -31 | -1 | Temporal_Mid_L | 21 | 5.23 |
|  | 29 | 18 | -97 | 17 | Occipital_Sup_R | 18 | 5.34 |
|  | 11 | -18 | -13 | -13 | Hippocampus_L | 28 | 5.13 |
|  |  | -15 | -22 | -16 | ParaHippocampal_L |  | 3.56* |
|  | 13 | -21 | -76 | -49 | Cerebelum_7b_L |  | 5.05 |
|  | 37 | 24 | -13 | -13 | Hippocampus_R |  | 3.79* |
|  |  | 18 | -10 | -19 | ParaHippocampal_R | 34 | 3.13* |
| RECO [Correct vs. Incorrect] | 4939 | -30 | -76 | -19 | Cerebellum_6_L | 19 | 15.14 |
|  |  | 24 | -82 | -16 | Fusiform_R |  | 13.39 |
|  |  | -33 | -70 | -16 | Fusiform_L |  | 12.97 |
|  |  | 21 | -76 | -10 | Lingual_R | 18 | 11.39 |
|  |  | -33 | -76 | -22 | Cerebellum_Crus1_L |  | 12.56 |
|  |  | 45 | -67 | -10 | Temporal_Inf_R |  | 9.17 |
|  |  | 12 | -94 | 23 | Cuneus_R | 19 | 5.43 |
|  |  | 21 | -49 | -22 | Cerebellum_4_5_R |  | 8.28 |
|  |  | -24 | -46 | -22 | Cerebellum_4_5_L |  | 7.56 |
|  |  | -42 | -49 | -16 | Temporal_Inf_L |  | 5.69 |
|  |  | -39 | -73 | -10 | Occipital_Inf_L |  | 12.35 |
|  |  | 39 | -73 | -13 | Occipital_Inf_R |  | 11.62 |
|  | 1979 | -24 | -55 | 53 | Parietal_Sup_L |  | 9.36 |
|  |  | -54 | -1 | 41 | Precentral_L | 6 | 8.85 |
|  |  | -54 | -16 | 41 | Postcentral_L | 4 | 6.05 |
|  |  | -54 | -25 | 44 | Parietal_Inf_L |  | 6.02 |
|  |  | -45 | 20 | 23 | Frontal_Inf_Tri_L |  | 6.1 |
|  |  | -45 | 8 | 26 | Frontal_Inf_Oper_L |  | 7.57 |
|  |  | -42 | 41 | 26 | Frontal_Mid_L |  | 5.36 |
|  |  | -24 | -4 | 56 | Frontal_Sup_L |  | 5.37 |
|  |  | -48 | -34 | 26 | SupraMarginal_L |  | 7.22 |
|  | 174 | 24 | -67 | 56 | Parietal_Sup_R | 7 | 8.35 |
|  |  | 30 | -67 | 32 | Occipital_Mid_R |  | 5.91 |
|  | 84 | 57 | 8 | 32 | Precentral_R |  | 7.52 |
|  |  | 45 | 8 | 26 | Frontal_Inf_Oper_R |  | 7.20 |
|  | 179 | 0 | 11 | 50 | Supp_Motor_Area_L |  | 7.31 |
|  |  | 6 | 14 | 50 | Supp_Motor_Area_R | 6 | 5.36 |
|  | 74 | -30 | -70 | -55 | Cerebelum_7b_L |  | 6.39 |
|  |  | -36 | -58 | -49 | Cerebelum_8_L |  | 6.34 |
|  | 11 | 42 | 23 | 17 | Frontal_Inf_Tri_R |  | 6.11 |
|  | 33 | 45 | 35 | 32 | Frontal_Mid_R |  | 6.01 |
|  | 26 | -30 | 20 | 5 | Insula_L |  | 5.76 |
|  | 36 | 36 | -4 | 65 | Frontal_Sup_R |  | 5.65 |
|  |  | 27 | 2 | 68 | Frontal_Sup_R |  | 5.63 |
|  |  | 42 | -1 | 56 | Frontal_Mid_R |  | 5.12 |
|  | 15 | 27 | 38 | -16 | Frontal_Mid_Orb_R |  | 5.38 |
|  | 37 | -21 | -1 | -16 | Amygdala_L |  | 4.12* |
|  |  | -18 | -13 | -13 | Hippocampus_L |  | 3.55* |
| RA  [Recall vs. Baseline] | 386 | -21 | -100 | 2 | Occipital_Mid_L |  | 11.75 |
|  |  | -24 | -88 | -4 | Occipital_Inf_L |  | 9.70 |
|  |  | -9 | -100 | -16 | Lingual_L |  | 9.08 |
|  |  | -27 | -85 | -19 | Cerebellum_Crus1_L |  | 7.52 |
|  | 260 | 24 | -94 | 8 | Occipital_Mid_R |  | 10.71 |
|  |  | 15 | -94 | -13 | Lingual_R | 17 | 6.4 |
|  |  | 27 | -79 | -19 | Cerebellum_6_R |  | 7.35 |
|  |  | 30 | -79 | -22 | Cerebellum_Crus1_R |  | 7.09 |
|  | 21 | -48 | -4 | 53 | Precentral_L | 6 | 7.00 |
|  | 69 | -63 | -25 | 2 | Temporal_Mid_L | 22 | 6.66 |
|  |  | -63 | -22 | 5 | Temporal_Sup_L |  | 5.71 |
|  | 66 | 60 | -19 | -1 | Temporal_Sup_R | 21 | 5.90 |
|  |  | 36 | -31 | -7 | Hippocampus_R |  | 3.52* |
|  | 43 | 51 | 14 | -13 | Temporal_Pole_Sup_R |  | 5.45 |
|  |  | 42 | 20 | -13 | Frontal_Inf_Orb_R | 47 | 5.03 |
|  | 24 | -57 | 8 | 17 | Frontal_Inf_Oper_L | 44 | 5.44 |
|  |  | -51 | 17 | 23 | Frontal_Inf_Tri_L |  | 5.01 |
|  | 13 | -60 | -37 | 17 | Temporal_Sup_L | 22 | 5.34 |
|  | 13 | -48 | -34 | 56 | Postcentral_L | 2 | 5.30 |
|  |  | -39 | -52 | 47 | Parietal_Inf_L | 40 | 4.71* |

* At p <.001 uncorrected

**Table S1.** Activated regions for one participant for Sentence generation, Encoding during sentence generation, Recognition of items and Recall. The number of voxels in the cluster (k), the x, y and z coordinates in millimetres, the anatomical region according to AAL atlas (Tzourio-Mazoyer et al., 2002), the Brodmann Area (BA) and the T value are indicated for each peak. All activations were obtained at *p* < .05 corrected except for those with asterisks in the table (**p* < .001 uncorrected). Abbreviation: GE = sentence Generation; ENCO = Encoding during sentence generation; RECO = Recognition of items; RA = Recall.
